# Impact of experience and specialization on accuracy and perceived performance of needling techniques in the cubital tunnel: a cadaveric study

**DOI:** 10.1007/s40477-026-01192-2

**Published:** 2026-07-29

**Authors:** Carlos López-de-Celis, Sergio Borrella-Andrés, Miguel Malo-Urriés, Isabel Albarova-Corral, Vanessa González-Rueda, Jacobo Rodríguez-Sanz

**Affiliations:** 1 ACTIUM Functional Anatomy Group Faculty of Medicine and Health Sciences, Universitat Internacional de Catalunya (UIC) Josep Trueta S/N, 08195 Sant Cugat del Vallés, Spain; 2 Department of Physiotherapy Faculty of Medicine and Health Sciences, Universitat Internacional de Catalunya (UIC) Josep Trueta S/N, 08195 Sant Cugat del Vallés, Spain; 3 Study Group On Pathology of the Locomotor System in Primary Care (GEPALAP), Institut Universitari d’Investigació en Atenció Primària (IDIAP Jordi Gol), 08007 Barcelona, Spain; 4 Healh Sciences Faculty, Department of Physiatry and Nursing, University of Zaragoza, 50009 Saragossa, Spain; 5 PhysiUZerapy Health Sciences Research Group, University of Zaragoza, 50009 Saragossa, Spain; 6 Department of Medicine Faculty of Medicine and Health Sciences, Universitat Internacional de Catalunya (UIC) Josep Trueta S/N, 08195 Sant Cugat del Vallés, Spain

**Keywords:** Ultrasound-guided needling, Palpation-guided needling, Invasive physiotherapy, Education, Cadaveric simulation, Professional experience, Perceived workload

## Abstract

**Background:**

Invasive needling procedures in physiotherapy can be performed using palpation or ultrasound guidance. While ultrasound offers greater precision and safety, the influence of healthcare professional experience and specialization in this field on performance has not yet been fully explored. The aim was to compare the accuracy, efficiency, safety, and perceived workload of ultrasound-guided versus palpation-guided needling procedures among physiotherapists with different levels of experience and specialization.

**Methods:**

Twenty-seven physiotherapists (students, non-specialized and specialized physiotherapists) performed 20 needling tasks each, on cadaveric specimens, targeting the ulnar nerve in the cubital tunnel. Procedures were conducted under two guidance conditions (ultrasound vs. palpation) and two handling modalities (with or without handpiece). Performance metrics (e.g., distance to target, structures affected) and subjective workload were recorded.

**Results:**

Specialized physiotherapists demonstrated significantly greater precision, efficiency, and safety than the other groups (*p* < 0.001). Students and non-specialized professionals had similar performance, particularly in terms of precision and control. Subjective workload was lower in specialists, especially under ultrasound guidance.

**Conclusion:**

Ultrasound guidance improves needling outcomes, especially when performed by trained specialists. General clinical experience without focused ultrasound training may be insufficient to ensure optimal procedural control. These findings support the integration of ultrasound training in advanced physiotherapy programs and the need to seek strategies that enhance learning.

## Introduction

Invasive physiotherapy techniques such as dry needling [1,2], percutaneous electrolysis, and other percutaneous interventions [3,4] are increasingly being integrated into clinical practice for the management of neuromusculoskeletal disorders [5,6]. Traditionally, these procedures have been performed using anatomical landmarks and palpation, relying heavily on the clinician's tactile and anatomical knowledge [7]. However, the advent of portable, high-resolution ultrasound devices has allowed for real-time visualization of deep structures, enhancing procedural accuracy and patient safety.

Ultrasound and echo-guided procedures are skills that are becoming increasingly common in healthcare practice [8–10]. Nevertheless, the learning curve [10–12] is recognized in relation to effort (time invested and repetitive practice) and knowledge acquisition [10]. Some guidelines establish minimum numbers of examinations performed to integrate competency, although based on expert consensus and not on solid data [8].

Ultrasound-guided needling has been associated with improved precision [13] and reduced complications, especially in anatomically complex or variable regions [14]. As a result, there has been a growing emphasis on incorporating sonographic skills into physiotherapy training [15,16]. Different studies on echo-guided procedures have established reference points from which one can categorize expertise [8], which would range between 30 and 75 procedures [4,6]. To try to accelerate this manual dexterity and shorten learning curves [17], simulators can be a good tool; nevertheless, expert supervision and structured feedback accelerate the learning curve and reduce technical errors [12,18] in ultrasound-guided procedures.

Nevertheless, not all practitioners receive structured ultrasound education, and many clinicians continue to rely solely on palpation-based techniques. This divergence in training raises the issue of whether general clinical experience can offset the absence of specific ultrasound guidance training when performing invasive procedures.

Despite the widespread adoption of ultrasound in physiotherapy, there is a lack of empirical evidence comparing how practitioners with different backgrounds (students, generalists, and specialists) perform under ultrasound-guided versus non-guided conditions. Moreover, no study to date has examined whether specialization in ultrasound-guided techniques results in measurable improvements in anatomical accuracy, technical efficiency, and perceived difficulty compared to general experience alone.

Therefore, the present study aims to analyze and compare the objective performance and subjective perception of physiotherapists with varying levels of experience when performing needling procedures guided either by ultrasound or palpation. Using a cadaveric simulation model, this research seeks to clarify the specific impact of specialization and training on procedural success, cognitive load, and user preference, thereby contributing to evidence-based recommendations for physiotherapy education and clinical practice.

The objective was to evaluate the accuracy, technical performance, and subjective perception of ultrasound-guided versus non-guided (palpation-based) needling techniques, applied by physiotherapists with different levels of training and experience.

## Methods

### Study design

This was a controlled, experimental, cross-sectional study conducted in a simulation environment. The study obtained the local Ethics Committee approval (CBAS-2021–09); Comitè d’Ética de Recerca of Universitat Internacional de Catalunya. This study conforms to all STROBE guidelines and reports the required information accordingly and complies with the Declaration of Helsinki (Brasil 2013) and Spanish regulations on research involving human anatomical specimens. All cadaveric limbs were provided through a university donation program with documented consent, and procedures were conducted respecting donor dignity and confidentiality. All physiotherapist participants provided written informed consent prior to the study, were informed of their right to withdraw at any time without consequences, and were assured of the confidentiality and anonymization of their data, in accordance with educational research ethical principles.

### Participants

Twenty-seven physiotherapists participated in the study and written informed consent was obtained from participants. The physiotherapists were stratified into three groups based on experience. Physiotherapy students without clinical experience (*n* = 8); Clinicians with general physiotherapy experience but no specialization in ultrasound-guided procedures (*n* = 9) and specialized physiotherapists with specific training in ultrasound-guided techniques (*n* = 10).

Their objective performance was evaluated through anatomical and procedural variables, and subjective perception was assessed via a post-task questionnaire.

### Samples

A cadaveric model was used to present a completely realistic situation in terms of anatomy. Frozen specimens were stored under refrigerated conditions (-20° C). To guarantee the normal characteristics of the tissue, the samples were thawed at room temperature 24 h before the procedure. Throughout the experimental sessions, limbs were kept hydrated using saline-soaked compresses.

Each cadaveric model was positioned similarly to the clinical situation with shoulder in slight abduction, elbow flexed at 90°, and the forearm supinated. This arrangement facilitated proper handling of the transducer and needle, replicating common clinical conditions.

Before starting the task series, all participants received a 10-min standardized instructional and hands-on session to ensure they understood the goals of the study and to allow them to become familiar with the materials, equipment, and needle manipulation under both guided and non-guided conditions [19].

### Needling procedure

Each participant performed needling tasks aiming to reach a defined target within the cubital tunnel, following anatomical and procedural criteria adapted from the methodology described by López-de-Celis et al. (2023) [20]. Specifically, participants were instructed to insert a needle as close as possible to the most superior and medial aspect of the ulnar nerve within the cubital tunnel, aiming for a proximity of less than 3 mm from the epineurium, while avoiding direct penetration of the nerve (target proximity task). The goal was to replicate a clinically relevant perineural approach, requiring fine anatomical discrimination and accurate motor control. Participants were allowed to make as many passes as necessary until they considered the final needle position to be optimal.

Each participant performed 20 invasive needling tasks, consisting of 10 ultrasound-guided procedures and 10 palpation-guided procedures. Within each guidance condition, five insertions were performed using a manipulator (handpiece), and five without, resulting in a 2 × 2 factorial design (guidance × manipulator use). The order of manipulator use was randomized using a computer-generated sequence. A washout break was allowed between each insertion, and a 5-min rest was scheduled after completing the first 10 tasks, to prevent the effect of fatigue on performance [21]. From a total of 540 potential punctures, 509 procedures were considered evaluable.

### Palpation-guided procedure

For the palpation-guided insertions, participants relied exclusively on their anatomical knowledge and palpatory skills. Using their dominant hand, the needle was inserted in the region between the medial epicondyle and the olecranon, after manually locating the course of the ulnar nerve within the cubital tunnel. Palpation-guided attempts were always performed before the ultrasound-guided trials to prevent visual learning bias from prior image interpretation [20].

### Ultrasound-guided procedure

In the ultrasound-guided condition, participants used a LOGIQ e R8 system (General Electric Healthcare) equipped with a 4–12 MHz linear transducer. All ultrasound parameters (depth, gain, frequency, and focal zone) were pre-calibrated by the research team to ensure image consistency across participants. The transducer was positioned transversely over the cubital tunnel, and the ulnar nerve was centered and visualized in short axis before proceeding to needle insertion. The needle was advanced from the medial aspect, using the dominant hand, while holding the probe in the non-dominant hand [20]. The predefined target corresponded to the most superficial and medial corner of the ulnar nerve epineurium within the cubital tunnel. Needle insertion was performed using an out-of-plane approach, and participants were instructed to position the needle tip as close as possible to the predefined target while avoiding nerve penetration.

**Fig. 1:**
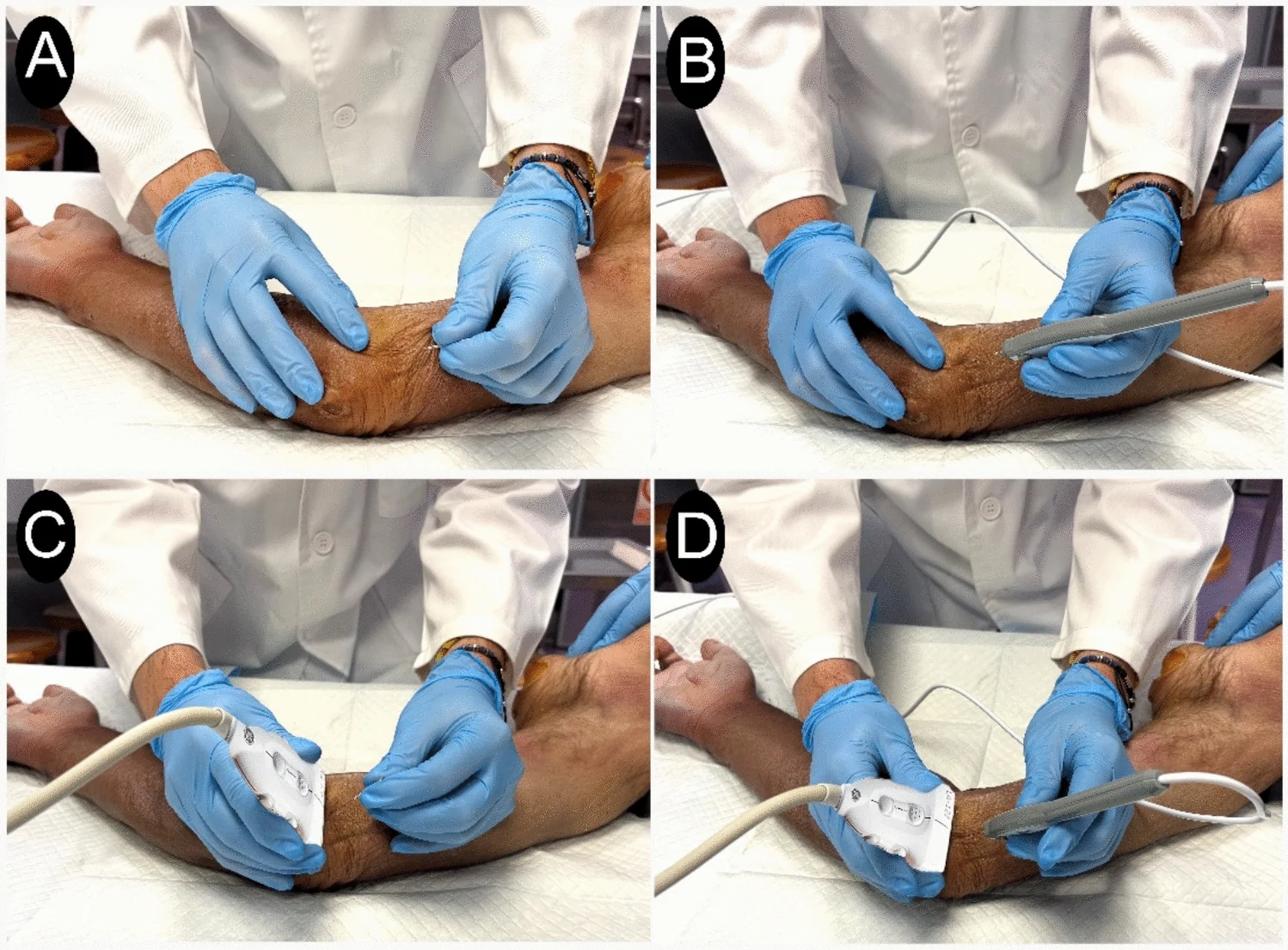
Procedure of the invasive techniques guided by palpation (**A** and **B**) and guided by ultra-sound imaging (**C** and **D**). The technique is performed without handpiece (**A** and **C**) and with a percutaneous electrolysis handpiece (**B** and **D**)

Both conditions used sterile 0.30 × 40 mm filiform needles (Agupunt, Barcelona), inserted either directly or through the standard percutaneous electrolysis handpiece. The use of the handpiece was included to simulate realistic clinical settings, particularly those involving electrotherapy-based techniques. (Fig. 1).

### Outcome assessment

#### Objective performance metrics

**Fig. 2:**
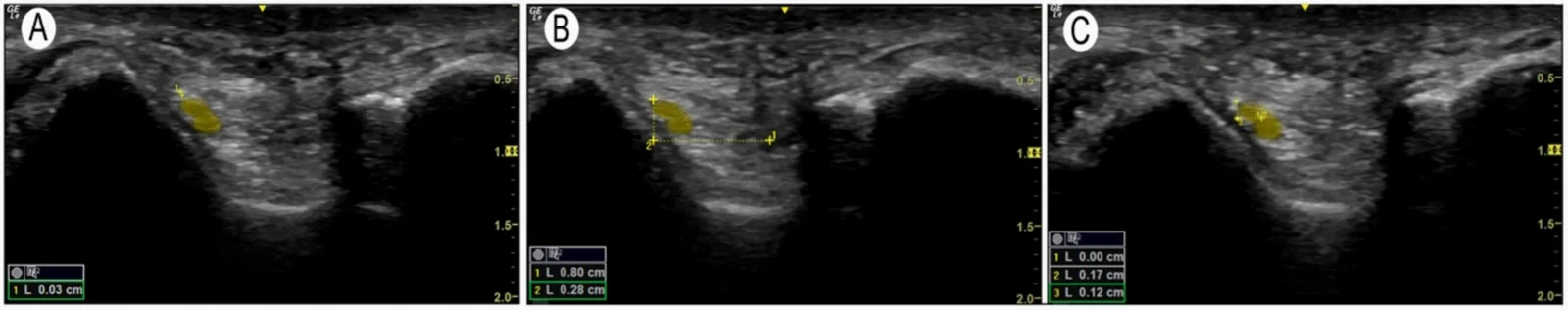
Representative ultrasound images illustrating different final needle positions relative to the ulnar nerve within the cubital tunnel. The predefined target corresponded to the most superficial and medial corner of the ulnar nerve epineurium within the cubital tunnel (Fig. 2). Two orthogonal measurements were obtained from the needle tip to the target point: an anteroposterior (AP) distance and a laterolateral (LL) distance. These measurements were subsequently used to calculate the absolute distance between the needle tip. **A** Optimal needle placement, with the needle tip positioned adjacent to the predefined target point located at the most superficial-medial aspect of the ulnar nerve epineurium, without nerve penetration. **B** Needle tip located outside the nerve and without penetration, but located more than 8 mm from the target. **C** Intraneural needle placement, with the needle tip penetrating the ulnar nerve

Target accuracy was assessed using ultrasound imaging. Following each procedure, the final needle position was independently evaluated by two experienced examiners who were blinded to the participant's professional group. The predefined target corresponded to the superficial-medial aspect of the ulnar nerve epineurium in the transverse ultrasound view (Fig. 2). Additionally, evaluators recorded whether the needle had penetrated the ulnar nerve, classifying such cases as intraneural placements. After each insertion, two experienced evaluators independently registered the following objective performance metrics [20]:

- Distance (mm) from the needle tip to the epineurium. The position of the needle tip relative to the target was determined using two orthogonal measurements: anteroposterior (AP) and laterolateral (LL) distance. These measurements were used to calculate the absolute distance between the needle tip and the predefined target point.
- Success/failure of the attempt. A successful procedure was defined as a final needle position located within 3 mm of the predefined target without intraneural penetration. Additionally, evaluators recorded whether the needle had penetrated the ulnar nerve, classifying such cases as intraneural placements.
- Total number of needle passes, defined as any redirection or advancement of the needle after initial insertion.
- Total procedure time (in seconds).
- Structures traversed, including any unintended tissue penetration.
- Length of exposed needle (mm) outside the cadaveric limb.

No feedback was given between tasks to avoid learning effects across trials, in line with prior studies showing that real-time feedback may improve short-term performance but introduces confounding effects in experimental designs [22,23].

#### Subjective Perception and Preferences

Upon completion of the 20 procedures, each participant completed a standardized questionnaire designed to assess perceived workload and cognitive demand. The questionnaire included mental, physical, and temporal demand, as well as perceived performance, effort, frustration, and self-rated ability [21], using 0–10-point Likert-type scales.

Finally, participants were asked to report their preferred needling approach (ultrasound-guided or palpation-guided and used of needle handpiece vs standard grip). Due to a technical error, the training data for two non-specialized physiotherapists and one specialized, along with the survey data from two non-specialized physiotherapists and two specialized, were lost.

### Statistical analysis

Statistical analyses were performed using IBM SPSS Statistics (v.29). Descriptive statistics included means and standard deviations for continuous variables, and frequencies and percentages for categorical variables, and were calculated for each variable across the three professional groups (students, non-specialized physiotherapists, and specialized physiotherapists) and for each guidance technique (palpation vs. ultrasound). Normality was assessed using the Shapiro–Wilk test. To evaluate the effect of professional experience, guidance technique, and their interaction on performance outcomes, we conducted a General Linear Model (Univariate GLM) for each dependent variable. This approach allows for simultaneous evaluation of main effects and interactions, while providing interpretable estimates of effect size (partial η^2^) and adjusted post hoc comparisons (Bonferroni correction). For the analysis of subjective perception variables, comparisons between groups were performed using the Kruskal–Wallis test for non-parametric continuous variables. This was followed by post-hoc pairwise comparisons using the Mann–Whitney U test. Categorical variables were analyzed using Fisher's exact test. Statistical significance was set at *p* < 0.05.

## Results

### Characteristics of the sample

The sample consisted of 11 women (40.7%) and 16 men (59.3%). Regarding professional background, the distribution was balanced among the three defined groups: students without clinical experience (29.6%), physiotherapists with general experience but without specialization (33.3%), and specialized physiotherapists (37.0%). The group of non-specialized physiotherapists has the highest average age at 38.22 years. (Table 1).

**Table 1:** General characteristics of the sample (n = 27)

Variables	n (%) or Mean ± SD
*Age*	
Student	25.63 ± 5.37
Non-specialized PTs	38.22 ± 12.18
Specialized PTs	29.75 ± 4.17
*Gender*	
Female	11 (40.7%)
Male	16 (59.3%)
*Professional group*	
Student	8 (29.6%)
Non-specialized PTs	9 (33.3%)
Specialized PTs	10 (37.0%)
*Education level*	
Student	8 (29.6%)
Graduate	19 (70.4%)

*n* number, *SD* Standard deviation, *%* percentage, *PTs* physiotherapist

Table 2 provides descriptive statistics on the participants’ previous training and professional experience by group. As expected, students reported no prior clinical experience or additional training hours. In contrast, non-specialized physiotherapists had an average of 8.86 ± 9.19 years working in the field, with 45.00 ± 36.17 h of specific dry needling training. Specialized physiotherapists presented the highest levels of experience and training, averaging 7.64 ± 3.70 years of practice and a median of 200 (60–200 IQR) hours of specific dry needling training. Weekly exposure to invasive techniques was highest in the specialized group, with a mean of 2.21 ± 0.99 weekly hours in dry needling technique and a median of 1 (IQR 1–3) weekly hours of ultrasound-guided practice application.

**Table 2:** Professional experience and prior training by group

Variabbles	Students	Non-specialized PTs	Specialized PTs
	Mean ± SD	Mean ± SD or Median (IQR)	Mean ± SD or Median (IQR)
Dry needling training (hrs)	0.00 ± 0.00	45.00 ± 36.17	200 (60–200)
Ultrasound training (hrs)	0.00 ± 0.00	0.00 ± 0.00	200 (45–200)
Ultrasound-guided training (hrs)	0.00 ± 0.00	0.00 ± 0.00	87.78 ± 47.38
Working PTs (yrs)	0.00 ± 0.00	8.86 ± 9.19	7.94 ± 3.83
Dry needling experience (yrs)	0.00 ± 0.00	4.71 ± 3.14	7.22 ± 4.41
Ultrasound experience (yrs)	0.00 ± 0.00	0.00 ± 0.00	6.78 ± 4.71
Ultrasound-guided experience (yrs)	0.00 ± 0.00	0.00 ± 0.00	4.39 ± 2.45
Dry needling practice—(weekly hrs)	0.00 ± 0.00	0.1 (0–1)	2.17 ± 0.87
Ultrasound practice—(weekly hrs)	0.00 ± 0.00	0.00 ± 0.00	2.55 ± 1.01
Ultrasound-guided practice—(weekly hrs)	0.00 ± 0.00	0.00 ± 0.00	1 (1–3)

*SD* standard deviation, *IQR* interquartile range, *PTs* physiotherapist

### Objective performance outcomes

#### Distance to target

Regarding the shortest distance between the needle tip and the target, there was a statistically significant interaction between group and technique (*F* = 4.846, *p* = 0.008, *η*^*2*^ = 0.019). A significant main effect was also observed for the group variable (*F* = 29.547, *p* < 0.001, *η*^*2*^ = 0.105), but not for the technique used (*F* = 2.288; *p* = 0.131; *η*^*2*^ = 0.005). (Table 3).

**Table 3:** Descriptive results and statistical comparisons by variable, group and guidance technique

Variable	Students			Non-specialized PTs			Specialized PTs		
	Mean ± SD	Difference between palpation and ultrasound-guided		Mean ± SD	Difference between palpation and ultrasound-guided		Mean ± SD	Difference between palpation and ultrasound-guided	
		Mean—95% CI	p-value		Mean—95% CI	p-value		Mean—95% CI	p-value
*Distance to target (mm)*									
Palpation-guided	4.15 ± 2.56	2.62 [0.17; 5.07]	0.036	6.86 ± 7.59	−1.88 [-4.09; 0.33]	0.095	3.42 ± 3.14	2.25 [0.20; 4.30]	0.031
Ultrasound-guided	1.54 ± 2.16			8.74 ± 15.37			1.17 ± 1.91		
*Time (s)*									
Palpation-guided	28.83 ± 12.58	−30.72 [-44.88; -16.56]	< 0.001	27.72 ± 18.13	−51.53 [-63.56; -39.50]	< 0.001	23.73 ± 20.37	−25.64 [-37.48; -13.79]	< 0.001
Ultrasound-guided	59.55 ± 39.30			79.25 ± 77.89			49.37 ± 44.18		
*Needle passes*									
Palpation-guided	3.47 ± 1.92	−1.24 [-3.10; 0.61]	0.189	2.16 ± 1.43	−6.95 [-8.53; -5.37]	< 0.001	2.12 ± 1.21	−0.16 [-1.71; 1.39]	0.840
Ultrasound-guided	4.71 ± 3.87			9.12 ± 12.45			2.28 ± 1.62		
*Structure length crossed (mm)*									
Palpation-guided	0.47 ± 0.82	0.30 [-0.14; 0.73]	0.178	0.95 ± 2.40	0.41 [0.04; 0.78]	0.028	0.47 ± 1.02	0.23 [-0.13; 0.60]	0.209
Ultrasound-guided	0.18 ± 0.63			0.53 ± 1.12			0.24 ± 0.73		
*Needle outside skin (mm)*									
Palpation-guided	17.49 ± 6.39	−1.44 [-3.10; 0.21]	0.088	16.36 ± 6.30	0.96 [-0.53; 2.44]	0.205	19.29 ± 3.40	0.99 [-2.45; 0.47]	0.185
Ultrasound-guided	18.93 ± 5.33			15.40 ± 4.24			18.30 ± 3.84		

*SD* standard deviation, *PTs* physiotherapist, *CI* confidence interval, *F* F-te;st, *η*^*2*^ eta-squared, *mm* millimeters, *s* seconds

In terms of group comparisons based on experience, we found that the most precise group was the specialized physiotherapists, who achieved a mean distance of 1.17 ± 1.91 mm to the target using the ultrasound-guided application. In contrast, the least precise group was the non-specialized physiotherapists using the same technique (8.74 ± 15.37 mm). (Table 3).

No significant differences were found between students and specialized physiotherapists for either application technique (*p* = 1.000). However, a significant difference was observed between students and non-specialized physiotherapists for the ultrasound-guided technique (p < 0.001), but not for the palpation-based puncture (*p* = 0.071). Finally, statistically significant differences were found between specialized and non-specialized physiotherapists for both application techniques. (Table 4).

**Table 4:** Bonferroni results and statistical comparisons by variable, group and guidance technique

Variable	Difference between students vs non-specialized PTs		Difference between students vs specialized PTs		Difference between non-specialized PTsvs specialized PTs	
	Mean—95% CI	p-value	Mean—95% CI	p-value	Mean -95% CI	p-value
*Distance to target (mm)*						
Palpation-guided	−2.71 [−5.58; 0.16]	0.071	0.74 [−2.03; 3.51]	1.000	3.44 [0.83; 6.06]	0.005
Ultrasound-guided	−7.21 [−10.05; -4.38]	< 0.001	0.37 [−2.39; 3.12]	1.000	7.58 [ 4.99; 10.17]	< 0.001
*Time (s)*						
Palpation-guided	1.12 [−14.91; 17.14]	1.000	5.10 [−10.86; 21.06]	1.000	3.99 [−10.57; 18.54]	1.000
Ultrasound-guided	−19.69 [−35.79; -3.60]	0.010	10.19 [-5.77; 26.15]	0.378	29.88 [15.25; 44.51]	< 0.001
*Needle passes*						
Palpation-guided	1.31 [−0.79; 3.41]	0.406	1.35 [−0.74; 3.44]	0.364	0.04 [−1.87; 1.95]	1.000
Ultrasound-guided	−4.40 [−6.52; −2.29]	< 0.001	2.43 [0.34; 4.53]	0.016	6.84 [4.91; 8.76]	< 0.001
*Structure length crossed (mm)*						
Palpation-guided	−0.47 [−0.96; 0.02]	0.066	0.00 [−0.49; 0.50]	1.000	0.48 [0.03; 0.92]	0.033
Ultrasound-guided	−0.36 [−0.85; 0.14]	0.254	−0.06 [−0.55; 0.43]	1.000	0.29 [−0.16; 0.74]	0.350
*Needle outside skin (mm)*						
Palpation-guided	1.13 [−0.79; 3.05]	0.473	−1.80 [−3.71; 0.10]	0.070	−2.93 [−4.73; −1.14]	< 0.001
Ultrasound-guided	3.53 [1.60; 5.46]	< 0.001	0.62 [−1.29; 2.54]	1.000	−2.90 [1.10; 4.71]	< 0.001

*PTs* physiotherapist, *CI* confidence interval, *mm* millimeters, *s* seconds

#### Application time

A statistically significant group-technique interaction was observed for the time variable (*F* = 4.956, *p* = 0.007; *η*^*2*^ = 0.018). A significant main effect was also noted for both the group variable (*F* = 7.778, *p* < 0.001; *η*^*2*^ = 0.029) and the technique used (*F* = 92.524, *p* = 0.001; *η*^*2*^ = 0.149). (Table 3).

Regarding speed, the fastest application was performed by the specialized physiotherapists using palpation-based puncture (23.73 ± 20.37 s). This group was also faster with the ultrasound-guided puncture (49.37 ± 44.18 s). In contrast, the slowest application was carried out by the non-specialized physiotherapists, also using the ultrasound-guided technique (79.25 ± 77.89 s). Across all groups, the time difference between palpation-based and ultrasound-guided puncture was greater for the ultrasound-guided application (*p* < 0.001). (Table 3).

No significant differences were found between students and specialized physiotherapists for either application technique (*p* > 0.378). However, a significant difference was observed between students and non-specialized physiotherapists for the ultrasound-guided technique (*p* < 0.010). The largest difference, a mean of 29.88 s, was found between specialized and non-specialized physiotherapists in the ultrasound-guided application (*p* < 0.001). (Table 4).

#### Needle passes

For the variable of needle passes, a statistically significant group-technique interaction was observed (*F* = 20.228, *p* < 0.001, *η*^*2*^ = 0.071). Significant main effects were also found for both the group variable (*F* = 18.666, *p* < 0.001, *η*^*2*^ = 0.066) and the technique used (*F* = 32.250; *p* < 0.001; *η*^*2*^ = 0.058). (Table 3).

Regarding the number of passes required, specialized physiotherapists performed the fewest passes for both techniques (palpation: 2.12 ± 1.21; ultrasound-guided: 2.28 ± 1.62). In contrast, non-specialized physiotherapists required the highest number of passes, particularly with the ultrasound-guided application (9.12 ± 12.45 passes) (Table 3). However, there was no significant difference in the number of passes for the palpation technique between specialized and non-specialized physiotherapists (*p* = 1.000). (Table 4).

#### Structure length crossed

There was no statistically significant Group × Technique interaction for the structure length crossed variable (*F* = 0.238, *p* = 0.788; *η*^*2*^ = 0.001). (Table 3).

#### Needle outside skin

There was no statistically significant Group × Technique interaction for the needle outside skin variable (*F* = 2.940, *p* = 0.054, *η*^*2*^ = 0.012). (Table 3).

#### Structure affected

In terms of safety and anatomical localization, the proportion of insertions without structural involvement was higher in the specialized group (92.0%), significantly superior to the non-specialized group (74.6%, *p* < 0.001) and to students (80.0%, *p* = 0.001 compared to the specialized group). Both students and non-specialized physiotherapists showed similar rates of unintended nerve contact, with no significant differences between them (*p* = 0.104). Non-specialized participants also showed occasional contact with the humeroulnar joint and triceps brachii, events not observed in the other groups. (Table 5).

**Table 5:** Descriptive and comparative analysis of performance metrics according to professional group

Variable	Students	Non-specialized PTs	Specialized PTs	
*Structure affected*				
None	112 (80%)	144 (74.6%)	184 (92%)	< 0.001*
Ulnar nerve	28 (20%)	41 (21.2%)	16 (8%)	
Humeroulnar joint	—	1 (0.5%)	—	
Triceps brachii	—	7 (3.6%)	—	
*Success/failure of the attempt*				
Yes	68 (48.9%)	38 (21.5%)	128 (64.3%)	< 0.001*
No	71 (51.1%)	139 (78.5%)	71 (35.7%)	

*SD* standard deviation, *PTs* physiotherapist, *mm* milimeters, *s* seconds, *Fisher's exact test

#### Success

A successful approach was defined as achieving an approximation of less than 3 mm without traversing another structure. Statistically significant differences were found between groups for this variable (*p* < 0.001). The most accurate group was the specialized physical therapists, with a 64.3% success rate. Non-specialized physical therapists were the least accurate, with a 78.5% failure rate, while students had similar percentages of success and failure (48.9% success, 51.1% failure). (Table 5).

#### Handpiece

No statistically significant difference was found for any variable within the Group × Handpiece interaction. This suggests that the use of the manipulator did not produce a statistically significant difference between manual and handpiece applications.

### Subjective perception and preferences

Regarding mental demand, no statistically significant differences were found during palpation-based procedures (*p* = 0.255). However, during ultrasound-guided techniques, a statistically significant group effect was observed (*p* = 0.015), as specialized physiotherapists reported substantially lower mental effort (2.00 ± 2.76) compared to non-specialized physiotherapists (7.33 ± 1.97) and students (5.43 ± 2.23). (Table 6).

**Table 6:** Descriptive and comparative analysis of subjective ratings and technique preferences by professional group

Variable	Students	Non-specialized PTs	Specialized PTs	p value
	Mean ± SD	Mean ± SD	Mean ± SD	
*Mental demand*				
Palpation-guided	5.14 ± 2.12	5.50 ± 3.62	2.67 ± 2.94	0.255*
Ultrasound-guided	5.43 ± 2.23	7.33 ± 1.97	2.00 ± 2.76	0.015*
*Physical demand*				
Palpation-guided	3.14 ± 2.04	5.00 ± 4.00	1.00 ± 1.27	0.078*
Ultrasound-guided	3.29 ± 2.43	6.00 ± 3.52	1.50 ± 2.35	0.085*
*Temporal demand*				
Palpation-guided	3.86 ± 2.80	4.50 ± 3.89	1.17 ± 1.33	0.120*
Ultrasound-guided	4.86 ± 2.03	4.67 ± 2.94	2.33 ± 2.73	0.205*
*Performance (self-rated)*				
Palpation-guided	4.57 ± 1.72	4.83 ± 2.79	5.67 ± 2.16	0.584*
Ultrasound-guided	7.71 ± 1.60	5.50 ± 2.81	9.17 ± 1.33	0.016*
*Effort*				
Palpation-guided	3.71 ± 2.87	3.67 ± 2.81	1.50 ± 1.64	0.222*
Ultrasound-guided	4.00 ± 2.65	5.50 ± 3.33	2.00 ± 2.76	0.183*
*Frustration*				
Palpation-guided	5.14 ± 3.53	3.17 ± 2.93	2.50 ± 2.74	0.300*
Ultrasound-guided	4.29 ± 2.43	5.50 ± 3.33	2.00 ± 1.90	0.105*
*Perceived skill*				
Palpation-guided	4.43 ± 2.15	5.00 ± 2.76	5.83 ± 2.48	0.497*
Ultrasound-guided	5.71 ± 2.14	3.50 ± 2.26	8.67 ± 1.51	0.005*
*Technique preferences—(n = 19 who answered this question (76% of the total)*				
*Guidance*				
Palpation-guided	0 (0%)	2 (33.3%)	0 (0%)	0.175**
Ultrasound-guided	7 (100%)	4 (66.7%)	6 (100%)	
*Use of handpiece*				
Without handpiece	5 (71.4%)	5 (83.3%)	6 (100%)	0.740**
With handpiece	2 (28.6%)	1 (16.7%)	0 (0%)	

*SD* standard deviation, *PTs* physiotherapist, *Kruskal–Wallis test, **Fisher's exact test test

Physical and temporal demands showed a trend toward lower values among specialized physiotherapists in both conditions, although these differences did not reach statistical significance. For example, during ultrasound-guided needling, specialized physiotherapists rated physical demand at 1.50 ± 2.35, versus 6.00 ± 3.52 in non-specialized physiotherapists (*p* = 0.085). Self-perceived performance also varied notably. While no statistically significant differences were observed with palpation (*p* = 0.584), specialized practitioners rated their ultrasound-guided performance significantly better (9.17 ± 1.33) than students (7.71 ± 1.60) and non-specialized physiotherapists (5.50 ± 2.81), with a statistically significant overall difference between groups (*p* = 0.016). (Table 6).

No statistically significant differences were found in effort or frustration levels, although specialists consistently reported lower scores in both domains. Similarly, perceived skill did not differ significantly under palpation (*p* = 0.497), but under ultrasound guidance, specialists perceived themselves as significantly more capable (8.67 ± 1.51; p = 0.005). (Table 6).

Finally, regarding technical preferences, the majority of participants preferred ultrasound guidance to palpation, although the difference was not statistically significant (*p* = 0.175). Interestingly, only two non-specialized physiotherapists preferred palpation, while all students and specialists chose ultrasound. Regarding the use of a handpiece, there was no significant difference in preference between groups (*p* = 0.740), although a small number of students and non-specialized physiotherapists opted for it. (Table 6).

Pairwise comparisons using the Mann–Whitney U test revealed a statistically significant difference between specialized and non-specialized physiotherapists for mental demand and performance (*p* = 0.012 and *p* = 0.008, respectively). Significant differences were also noted between students and specialized physiotherapists in mental demand and temporal demand (*p* = 0.035 and *p* = 0.049, respectively).

## Discussion

This study aimed to assess the accuracy, performance, and perceived workload of ultrasound-guided versus palpation-guided needling procedures among physiotherapists with different levels of experience and training. By simulating a realistic cadaveric model, we were able to isolate and analyze key differences in performance and perceptions across three groups: students without clinical experience, non-specialized physiotherapists, and specialized physiotherapists. Our results show clear differences in performance across professional levels. Specialized physiotherapists demonstrated superior accuracy (shorter distance to the target), greater efficiency (fewer needle passes, shorter time), and improved safety (lower incidence of unintended structure penetration). In contrast, non-specialized physiotherapists, despite more years in the profession than students, often performed similarly to students, especially in terms of precision and control. Subjective perceptions also followed this trend, with specialists reporting lower mental and physical demand and higher self-rated performance under ultrasound-guided conditions.

### Objective performance

The most consistent and significant differences were observed in variables directly linked to procedural accuracy. Specialized physiotherapist achieved closer proximity to the target, minimized unnecessary tissue traversal, and avoided critical structures such as the ulnar nerve more reliably than other groups. Notably, students reached similar levels of contact area to specialized physiotherapist in some cases, possibly reflecting the benefits of focused, recent training even in inexperienced practitioners.

Interestingly, non-specialized physiotherapist did not consistently outperform students, particularly in terms of number of passes or external needle length. This may suggest that professional experience alone, without specialization or focused ultrasound training, is insufficient to enhance invasive procedural control.

Regarding distance to target, a statistically significant difference was found in the group-by-technique interaction (*p* = 0.008), without observing a significant main effect for technique alone (*p* = 0.131). This indicates that the ultrasound-guided approach improved performance in students and specialized physiotherapists, but did not benefit and even worsened performance in non-specialized professionals. This differential response suggests that the ultrasound-guided approach does not translate into better results without adequate training. In the case of students, having no training in either of the two proposed techniques did not generate cognitive overload as we see with similar mental demand between techniques (Table 6), which perhaps generated greater tranquility during execution and translated into greater precision. The inability of the non-specialized group to effectively leverage ultrasound guidance may be due to cognitive overload when simultaneously managing probe placement, image interpretation, and needle manipulation without adequate preparation, as corroborated in Table 6 where the mental demand of ultrasound-guided use by non-specialized physiotherapists was the highest.

Regarding execution time, it was observed that execution time is greater with the ultrasound-guided approach (*p* < 0.001), similar to findings in the previous study by López-de-Celis et al. (2023) [20]. Specialists demonstrated superior temporal control (49.4 s). The significantly increased number of needle passes with ultrasound guidance, especially among non-specialized physiotherapists, suggests that visual feedback encourages perfectionist tendencies and multiple corrections. Conversely, palpation-guided procedures result in fewer corrective maneuvers due to lack of feedback. The consistent maintenance of a low number of needle passes by specialized physiotherapists, regardless of guidance method, demonstrates that advanced training develops both technical competence and appropriate clinical judgment regarding procedural endpoints.

Regarding the structure length crossed variable evaluated in millimeters, this was consistently greater with the palpation approach compared to ultrasound-guided in all groups. This clearly indicates a general safety benefit of visual guidance.

No statistically significant difference was observed in handpiece use between groups, similar to previous studies [14,20], suggesting effective normalization of ergonomic design.

Our findings are consistent with previous studies indicating that ultrasound guidance enhances the precision and safety of needling techniques in both clinical and educational settings [24–27]. Similar to López-de-Celis et al. (2023) [20], we found that real-time visualization aids both targeting accuracy and confidence. However, while previous literature often groups professionals by years of experience, our study uniquely distinguishes between non-specialized and specialized profiles, revealing a clear benefit of advanced, focused training.

### Subjective perception and preferences

Specialized professionals consistently reported lower perceived workload and higher confidence during ultrasound-guided procedures. Differences in mental demand and perceived skill were statistically significant in this condition, confirming that training and familiarity with ultrasonography reduce cognitive load and increase confidence.

Preference analysis revealed that most participants, regardless of group, favored ultrasound guidance over palpation, although this did not reach statistical significance. Only non-specialized physiotherapist reported a small proportion preferring palpation, possibly reflecting resistance to technological reliance in this subgroup.

The use of the handpiece did not significantly affect preferences across groups. This might indicate that comfort with the tool requires more familiarization or that its added benefit was not perceived clearly in this experimental context.

### Educational implications for improving eco-guided learning in physiotherapy

The results of our study highlight the imperative need for specialized and structured ultrasound education in physiotherapy, as general clinical experience is insufficient to ensure proficiency in ultrasound-guided invasive procedures. The integration of ultrasound in advanced physiotherapy programs is crucial, since specialists demonstrated greater precision, efficiency, and safety.

To accelerate the learning curve, we believe it is fundamental to incorporate expert supervision and structured feedback [12,18]. Deliberate practice under the guidance of experienced professionals could optimize the development of handling skills and image interpretation.

Likewise, the use of simulation models, both with the implementation of phantoms[28–30] and cadaveric specimens, is revealed as a valuable educational tool. It enables repetitive and safe practice, essential for achieving the necessary competence [4,6,8], and facilitates familiarization with anatomy and manipulation dexterity.

Given that general experience does not compensate for the lack of ultrasound training, it is vital to develop continuing education programs specific for practicing physiotherapists. These should focus not only on technique, but also on reducing cognitive load and increasing professional self-confidence, aspects that improve significantly with ultrasound specialization.

### Regulatory context and professional development

The resolution of the General Council of Physiotherapy Colleges of Spain dated 19-11-2011 (nº-5/2001) approves the competence for dry needling application by physiotherapists, without specifying whether this should or should not be performed in combination with ultrasound guidance [31]. The use of ultrasound by physiotherapists is supported by resolution nº-1/2023, of the General Council of Physiotherapy Colleges of Spain dated 29-03-2023 [31]. Therefore, it is relatively recent, despite current students considering these techniques as integral components of the curriculum. However, non-specialized physiotherapists with longer career trajectories lack formal experience, which contributes to the performance disparities observed.

Invasive physiotherapy requires the implementation of training programs to bridge knowledge gaps. There are increasingly more official university training programs in invasive physiotherapy verified by official bodies such as the National Agency for Quality Assessment and Accreditation (ANECA).

The findings of this study suggest that ultrasound-guided invasive techniques should be considered advanced skills requiring specific training, rather than assuming that clinical experience can substitute for them. Ultrasound training programs should be prioritized, and consideration should be given to the fact that the ultrasound-guided approach produces greater healthcare benefits.

### Limitations

This study has several limitations. First, it was conducted in a simulated cadaveric environment, which, although highly realistic, cannot fully replicate patient-specific anatomical variations or clinical conditions. Second, the sample size, especially for subjective preference data, was limited, as only 76% of participants completed preference questions. Another limitation is that target accuracy was assessed using ultrasound imaging rather than systematic anatomical dissection. Although this approach allowed preservation of the cadaveric specimens and enabled evaluation of a large number of procedures, minor measurement errors inherent to ultrasound-based assessment cannot be completely excluded. Additionally, the use of a cross-sectional design does not allow for evaluation of learning curves or the retention of skills over time.

Future studies should explore longitudinal training effects, include larger and more diverse samples, and consider real-patient application outcomes. Investigating whether tailored educational interventions based on baseline experience or confidence could accelerate skill acquisition may also be valuable.

## Conclusion

Ultrasound-guided needling procedures result in superior accuracy, efficiency, and safety when compared to palpation-guided approaches, particularly when performed by specialized physiotherapists. These professionals also report lower cognitive and physical demand and greater perceived performance, supporting the integration of ultrasound training in advanced physiotherapy education. Conversely, years of general experience without specialization did not necessarily translate into better procedural outcomes. Our results highlight the importance of structured ultrasound training and suggest that even novice practitioners may achieve high performance with appropriate guidance and simulation practice.

## Data Availability

Harvard dataverse: https://doi.org/10.7910/DVN/LTS76O.
